# Impact of Intramyocardial Hemorrhage on Clinical Outcomes in ST-Elevation Myocardial Infarction: A Systematic Review and Meta-analysis

**DOI:** 10.1016/j.jscai.2022.100444

**Published:** 2022-08-26

**Authors:** Rohit Vyas, Khalid H. Changal, Sapan Bhuta, Vanessa Pasadyn, Konrad Katterle, Matthew J. Niedoba, Keyur Vora, Rohan Dharmakumar, Rajesh Gupta

**Affiliations:** a Division of Cardiovascular Medicine, University of Toledo, Toledo, Ohio; b Department of Internal Medicine, University of Toledo, Toledo, Ohio; c Department of Medicine and Cardiovascular Institute, Krannert Cardiovascular Research Center, Indiana University School of Medicine, Indianapolis, Indiana

**Keywords:** adverse remodeling, intramyocardial hemorrhage, MACE, microvascular obstruction, myocardial infarction, STEMI

## Abstract

**Background:**

Intramyocardial hemorrhage (IMH) occurs after ST-elevation myocardial infarction (STEMI) and has been documented using cardiac magnetic resonance imaging. The prevalence and prognostic significance of IMH are not well described, and the small sample size has limited prior studies.

**Methods:**

We performed a comprehensive literature search of multiple databases to identify studies that compared outcomes in STEMI patients with or without IMH. The outcomes studied were major adverse cardiovascular events (MACE), infarct size, thrombolysis in myocardial infarction (TIMI) flow after percutaneous coronary intervention (PCI), left ventricular end-diastolic volume (LVEDV), left ventricular ejection fraction (LVEF), and mortality. Odds ratios (ORs) and standardized mean differences with corresponding 95% CIs were calculated using a random effects model.

**Results:**

Eighteen studies, including 2824 patients who experienced STEMI (1078 with IMH and 1746 without IMH), were included. The average prevalence of IMH was 39%. There is a significant association between IMH and subsequent MACE (OR, 2.63; 95% CI, 1.79-3.86; *P* < .00001), as well as IMH and TIMI grade <3 after PCI (OR, 1.75; 95% CI, 1.14-2.68; *P* = .05). We also found a significant association between IMH and the use of glycoprotein IIb/IIIa inhibitors (OR, 2.34; 95% CI, 1.42-3.85; *P* = .0008). IMH has a positive association with infarct size (standardized mean difference, 2.19; 95% CI, 1.53-2.86; *P* < .00001) and LVEDV (standardized mean difference, 0.7; 95% CI, 0.41-0.99; *P* < .00001) and a negative association with LVEF (standardized mean difference, −0.89; 95% CI, −1.15 to −0.63; *P* = .01). Predictors of IMH include male sex, smoking, and left anterior descending infarct.

**Conclusions:**

Intramyocardial hemorrhage is prevalent in approximately 40% of patients who experience STEMI. IMH is a significant predictor of MACE and is associated with larger infarct size, higher LVEDV, and lower LVEF after STEMI.

## Highlights

• Intramyocardial hemorrhage is present in approximately 40% of reperfused patients who experienced ST-elevation myocardial infarction.
• Intramyocardial hemorrhage is associated with major adverse cardiovascular events after ST-elevation myocardial infarction.
• Intramyocardial hemorrhage is associated with larger infarct size, lower ejection fraction, and larger left ventricular end-diastolic volume after ST-elevation myocardial infarction.
• Intramyocardial hemorrhage is associated with more frequent thrombolysis in myocardial infarction flow score of <3 after percutaneous coronary intervention and use of GP IIb/IIIa inhibitors.
• Predictors of intramyocardial hemorrhage after ST-elevation myocardial infarction are male sex, left anterior descending infarct, and smoking.

## Central Illustration

**Figure:**
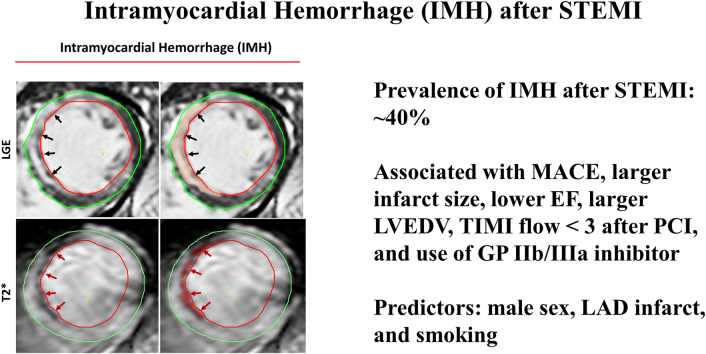

## Introduction

Early reperfusion therapy has been established as the standard of care in managing acute ST-segment elevation myocardial infarction (STEMI).^1^ The last several decades have seen significant improvement in mortality rates for patients with STEMI. However, the success of reperfusion therapy is frequently limited by failed tissue perfusion.^2^ Microvascular obstruction (MVO) and intramyocardial hemorrhage (IMH) are 2 pathologies underlying failed tissue perfusion after the restoration of epicardial coronary blood flow. IMH reflects the aggregation and extravasation of erythrocytes and is a manifestation of severe microvascular injury.^3^^,^^4^ The extent of hemorrhage after myocardial infarction is influenced by the duration of ischemia, reperfusion, and severity of STEMI.^5^

**Figure 1:**
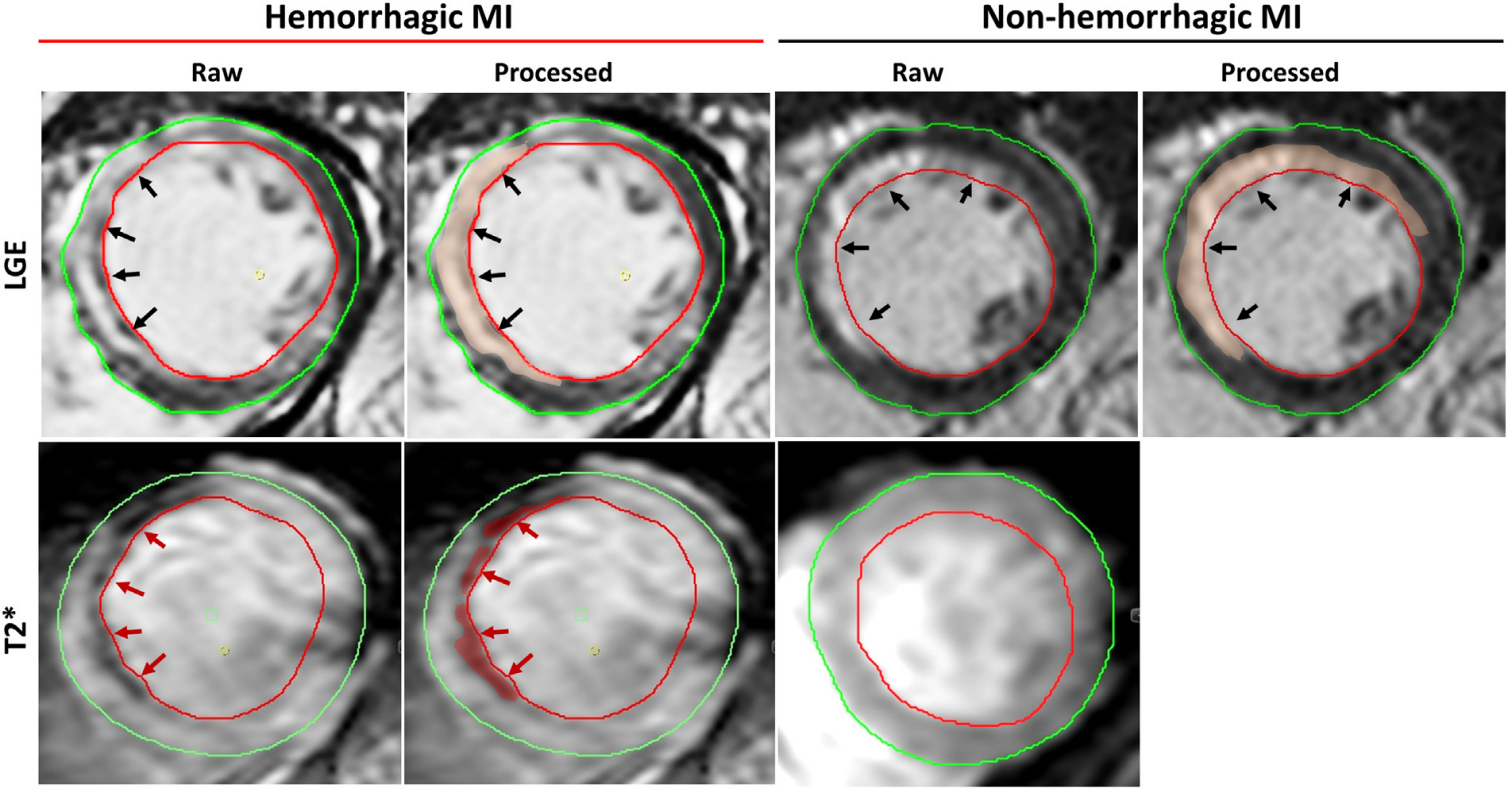
**Representative cardiovascular magnetic resonance (CMR) images of intramyocardial hemorrhage (IMH).** LGE, late gadolinium enhancement; MI, myocardial infarction; T2∗, T2-weighted images with multiple echo times.

In recent years, multiple smaller observational clinical studies have investigated whether contrast-enhanced cardiovascular magnetic resonance (CMR) allows for accurate assessment of IMH and MVO in patients who experience STEMI and whether their presence has prognostic relevance. IMH can be detected via T2- and T2∗-weighted CMR approaches based on the paramagnetic properties of hemoglobin breakdown products (Figure 1).^6^

A limited number of studies have evaluated the prognostic significance of IMH in the setting of the acute coronary syndrome. Although the clinical significance of MVO has been well characterized,^7^ a better understanding of the clinical and prognostic significance of IMH is needed. Furthermore, a recent study has convincingly demonstrated that IMH is not simply a marker of large infarcts but is an actual biological driver of infarct expansion.^8^ This study aimed to conduct a systematic review and meta-analysis to determine the prevalence of IMH after STEMI and whether IMH is associated with adverse outcomes after STEMI.

## Methods

**Figure 2:**
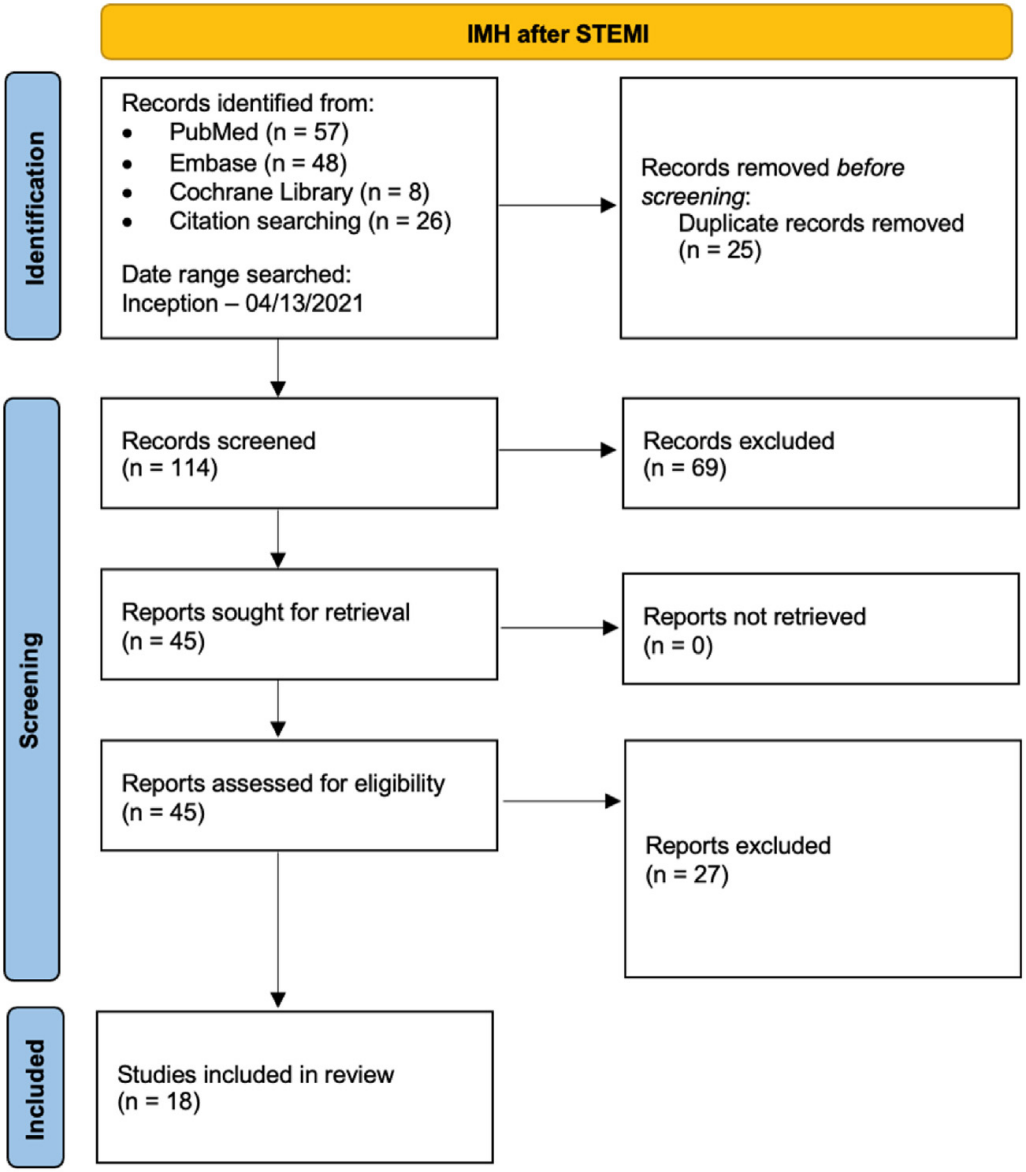
**Preferred Reporting Items for Systematic Reviews and Meta-Analyses (PRISMA) flow diagram for the selection of studies.** IMH, intramyocardial hemorrhage; STEMI, ST-elevation myocardial infarction.

This systematic review was conducted according to the Preferred Reporting Items for Systematic Reviews and Meta-Analyses guidelines.^9^ The search strategy is provided in Figure 2 and the Supplemental File. The database was searched from inception to April 2021.

We included randomized controlled trials, cohort studies, case-control studies, and case series of patients who experienced STEMI who had IMH assessed with an imaging study. We excluded animal studies, case reports, case series with a sample size of <10 patients, reviews, editorials, letters to editors, and studies with incomplete data. Two investigators (R.V. and K.H.C.) independently screened and selected the studies for the final review. Differences in opinion were resolved with mutual discussion and adjudication by the senior author (R.G.). Demographic data and study characteristics were extracted. The outcomes studied were major adverse cardiovascular events (MACE), all-cause mortality, infarct size, left ventricular end-diastolic volume (LVEDV), left ventricular ejection fraction (LVEF), and presence of thrombolysis in myocardial infarction (TIMI) flow score of <3 after percutaneous coronary intervention (PCI).

We used the random effects model of DerSimonian and Laird to calculate the aggregated odds ratio (OR) and corresponding 95% confidence interval (CI) for MACE, all-cause mortality, and TIMI-3 flow after PCI. Heterogeneity was assessed using the Higgins I2 statistic, with values <25% and >75% considered indicative of low and high heterogeneity, respectively. Publication bias was assessed visually by the asymmetry in funnel plots (Supplemental File).

Standardized mean difference (SMD) was used for effect size measurement to evaluate LVEDV, LVEF, and infarct size with a 95% CI. The effect size of 0.2 to 0.5 is considered small, 0.5 to 0.8 is medium, and >0.8 is large. We further performed subgroup analyses to specifically evaluate the effect of T2∗ magnetic resonance imaging (MRI) versus standard T2 MRI on the specified outcomes, as these modalities have been shown to differ in their ability to identify IMH.

All tests were 2-tailed, with a *P* value of <.05 considered statistically significant. Statistical analyses were performed using Review Manager version 5.3 (The Nordic Cochrane Center, The Cochrane Collaboration, 2014), R version 3.6.2, and the Meta-Essentials tool (Erasmus Research Institute of Management).

## Results

### Study selection

Our search strategy retrieved a total of 114 studies. Among these, 45 were eligible for systematic review. Subsequently, we excluded 27 studies because these were either duplicate, did not use cardiac MRI, or did not report outcome data. Eventually, 18 studies met our inclusion criteria and were included in the meta-analysis.^10^, ^11^, ^12^, ^13^, ^14^, ^15^, ^16^, ^17^, ^18^, ^19^, ^20^, ^21^, ^22^, ^23^, ^24^, ^25^, ^26^, ^27^, ^28^ Figure 2 shows the Preferred Reporting Items for Systematic Reviews and Meta-Analyses flowchart that illustrates how the final studies were selected.

### Study characteristics

**Figure 3:**
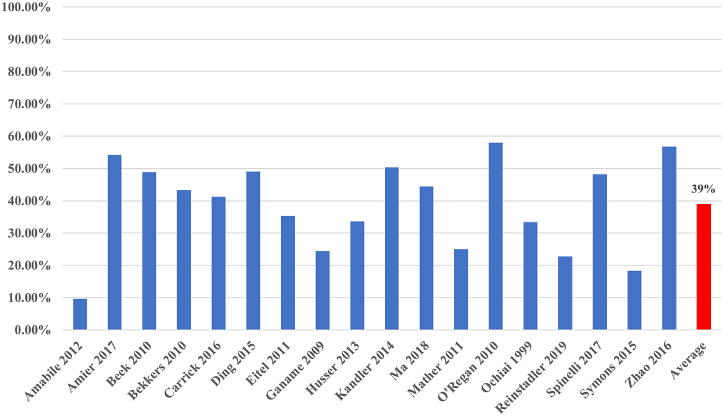
**Prevalence of intramyocardial hemorrhage in ST-elevation myocardial infarction.**

Table 1 shows the baseline characteristics of the studies included in the meta-analysis. All 18 studies were published between 1999 and 2019. A total of 2824 patients were included from these studies. Overall, 39% of these patients were found to have IMH (Figure 3). The mean age of the patients was 59.2 years, and men represented 74.9% of the total patients. Among all included studies, CMR was performed over a range of 1 to 8 days after STEMI, with 1 study performing CMR up to 14 days after STEMI. The vast majority of patients with IMH also had coexisting MVO. IMH was significantly associated with male sex, smoking, and left anterior descending (LAD) infarcts. No association was seen between IMH and diabetes, hypertension, hyperlipidemia, and non-LAD infarcts (Table 2). We assessed the quality of the included studies using the Newcastle-Ottawa scale for observational studies as shown in Supplemental Table S1.

**Table 1:** Baseline comorbidities and patient population characteristics.

Reference, year	MRI protocol	Subgroup	Number of patients	Time to MRI (d)^a^^,^^b^	Age (y)	Male sex	BMI (kg/m^2^)	Diabetes^c^	Hyper-cholesterolemia^c^	Hypertension^c^	Smoking^c^
Amabile et al,^10^ 2012	T2-weighted	No IMH	103	5.5 ± 3.4^a^	57.5	85 (82.5%)	26.6	16 (15.5%)	35 (34.0%)	38 (36.9%)	63 (61.2%)
		IMH	11	4.7 ± 1.9^a^	58.3	10 (90.9%)	26.5	3 (27.3%)	4 (36.4%)	5 (45.5%)	5 (45.5%)
Amier et al,^12^ 2017	T2-weighted	No IMH	188	5.5 ± 1.8^a^	59.5	148 (78.7%)	27	29 (15.4%)	61 (32.4%)	85 (45.2%)	144 (76.6%)
		IMH	222		57.7	195 (87.8%)	27.2	31 (14.0%)	82 (36.9%)	78 (35.1%)	174 (78.4%)
Beek et al,^13^ 2010	T2-STIR	No IMH	23	4.3 ± 2.1^a^	59	21 (91.3%)	-	2 (8.7%)	5 (21.7%)	6 (26.1%)	14 (60.9%)
		IMH	22	5.7 ± 2.0^a^	54	19 (86.4%)	-	0 (0%)	8 (36.4%)	6 (27.3%)	14 (63.6%)
Bekkers et al,^14^ 2010	T2-STIR	No IMH	51	5 ± 2 a	60.6	33 (64.7%)	-	5 (9.8%)	14 (27.5%)	23 (45.1%)	42 (82.4%)
		IMH	39		59	32 (82.1%)	-	1 (2.6%)	11 (28.2%)	12 (30.8%)	36 (92.3%)
Carrick et al,^15^ 2016	T2∗-weighted	No IMH	144	2.1 ± 1.8^a^	58.6	103 (71.5%)	27.8	13 (9.0%)	37 (25.7%)	40 (27.8%)	83 (57.6%)
		IMH	101		59	84 (83.2%)	28	15 (14.9%)	31 (30.7%)	37 (36.6%)	70 (69.3%)
Ding et al,^16^ 2015	T2-STIR	No IMH	55	5^b^	59.2	47 (85.5%)	-	25 (45.5%)	28 (50.9%)	32 (58.2%)	40 (72.7%)
		IMH	53		57.6	43 (81.1%)	-	14 (26.4%)	31 (58.5%)	26 (49.1%)	41 (77.4%)
Eitel et al,^17^ 2011	T2-STIR	No IMH	224	3^b^	65	162 (72.3%)	-	49 (21.9%)	81 (36.2%)	146 (65.2%)	84 (37.5%)
		IMH	122		62	94 (77.0%)	-	29 (23.8%)	40 (32.8%)	76 (62.3%)	56 (45.9%)
Ganame et al,^11^ 2009	T2-STIR	No IMH	74	2^b^	60	60 (81.1%)	-	7 (9.5%)	38 (51.4%)	19 (25.7%)	33 (44.6%)
		IMH	24		57.7	24 (100.0%)	-	3 (12.5%)	16 (66.7%)	8 (33.3%)	16 (66.7%)
Husser et al,^18^ 2013	T2-STIR	No IMH	202	6^b^	59	163 (80.7%)	-	28 (13.9%)	79 (39.1%)	92 (45.5%)	114 (56.4%)
		IMH	102		57	81 (79.4%)	-	25 (24.5%)	37 (36.3%)	49 (48.0%)	68 (66.7%)
Kandler et al,^19^ 2014	T2∗-weighted	No IMH	75	2.9^b^	62	53 (70.7%)	28.7	14 (18.7%)	32 (42.7%)	51 (68.0%)	37 (49.3%)
		IMH	76		60	61 (80.3%)	27.9	18 (23.7%)	20 (26.3%)	52 (68.4%)	44 (57.9%)
Ma et al,^20^ 2018	T2∗-weighted	No IMH	35	3.0 ± 1.1^a^	56	-	24.8	7 (20.0%)	4 (11.4%)	19 (54.3%)	27 (77.1%)
		IMH	28		51.5	-	25.5	3 (10.7%)	9 (32.1%)	10 (35.7%)	23 (82.1%)
Mather et al,^21^ 2011	T2- and T2∗-weighted	No IMH	36	2^b^	57.5	32 (88.9%)	-	1 (2.8%)	19 (52.8%)	12 (33.3%)	19 (52.8%)
		IMH	12		56	11 (91.7%)	-	3 (25.0%)	8 (66.7%)	4 (33.3%)	6 (50.0%)
O’Regan et al,^27^ 2010	T2∗-weighted	No IMH	21	3.1 ± 2.0^a^	54	20 (95.2%)	-	1 (4.8%)	-	6 (28.6%)	15 (71.4%)
		IMH	29		55	28 (96.6%)	-	6 (20.7%)	-	8 (27.6%)	14 (48.3%)
Ochiai et al,^28^ 1999	T2∗-weighted	No IMH	26	5.7^b^	63	17 (65.4%)	-	-	-	-	-
		IMH	13		63	7 (53.8%)	-	-	-	-	-
Reinstadler et al,^22^ 2019	T2∗-weighted	No IMH	204	3^b^	61	131 (64.2%)	28	32 (15.7%)	78 (38.4%)	130 (63.7%)	98 (49.2%)
		IMH	60		59	44 (73.3%)	26	7 (11.7%)	17 (28.3%)	37 (61.7%)	26 (45.6%)
Spinelli et al,^24^ 2018	T2-STIR/speckle-tracking echocardiography analysis	No IMH	45	-	58.6	39 (86.7%)	-	13 (28.9%)	22 (48.9%)	22 (48.9%)	26 (57.8%)
		IMH	42		59.3	38 (90.5%)	-	14 (33.3%)	19 (45.2%)	20 (47.6%)	23 (54.8%)
Symons et al,^25^ 2015	T2-STIR	No IMH	152	2.9^b^	59	123 (80.9%)	-	-	-	46 (30.3%)	-
		IMH	34		59	32 (94.1%)	-	-	-	14 (41.2%)	-
Zhao et al,^26^ 2016	T2-STIR	No IMH	35	8^b^	57.7	33 (94.3%)	-	14 (40.0%)	13 (37.1%)	19 (54.3%)	14 (40.0%)
		IMH	46		57	43 (93.5%)	-	18 (39.1%)	30 (65.2%)	26 (56.5%)	18 (39.1%)

BMI, body mass index; IMH, intramyocardial hemorrhage; MRI, magnetic resonance imaging; T2-STIR, T2-weighted imaging with short-tau inversion recovery magnetization preparation.

a Values are reported as mean ± SD number of days.

b Values are reported as median number of days.

c Values expressed as n (%).

**Table 2:** Predictors of intramyocardial hemorrhage after ST-elevation myocardial infarction.

Baseline characteristics	IMH	No IMH	Studies	OR (95% CI)	Heterogeneity	
					*P* value	I^2^
Male	814	1147	16	2.10 (1.72-2.55)	.00001	79
Diabetes	190	275	16	0.99 (0.80-1.22)	.9	58
Hypertension	454	740	16	0.91 (0.77-1.08)	.28	0
Smoking	634	853	16	1.24 (1.04-1.48)	.02	0
Hyperlipidemia	363	546	15	1.07 (0.90-1.27)	.46	37
LAD artery infarct	270	279	9	2.51 (1.95-3.24)	.00001	62
Non-LAD artery infarct (LCx artery or RCA)	154	386	8	0.44 (0.33-0.58)	.00001	21

IMH, intramyocardial hemorrhage; LAD, left anterior descending; LCx, left circumflex; OR, odds ratio; RCA, right coronary artery.

### Association of IMH with clinical outcomes

**Figure 4:**
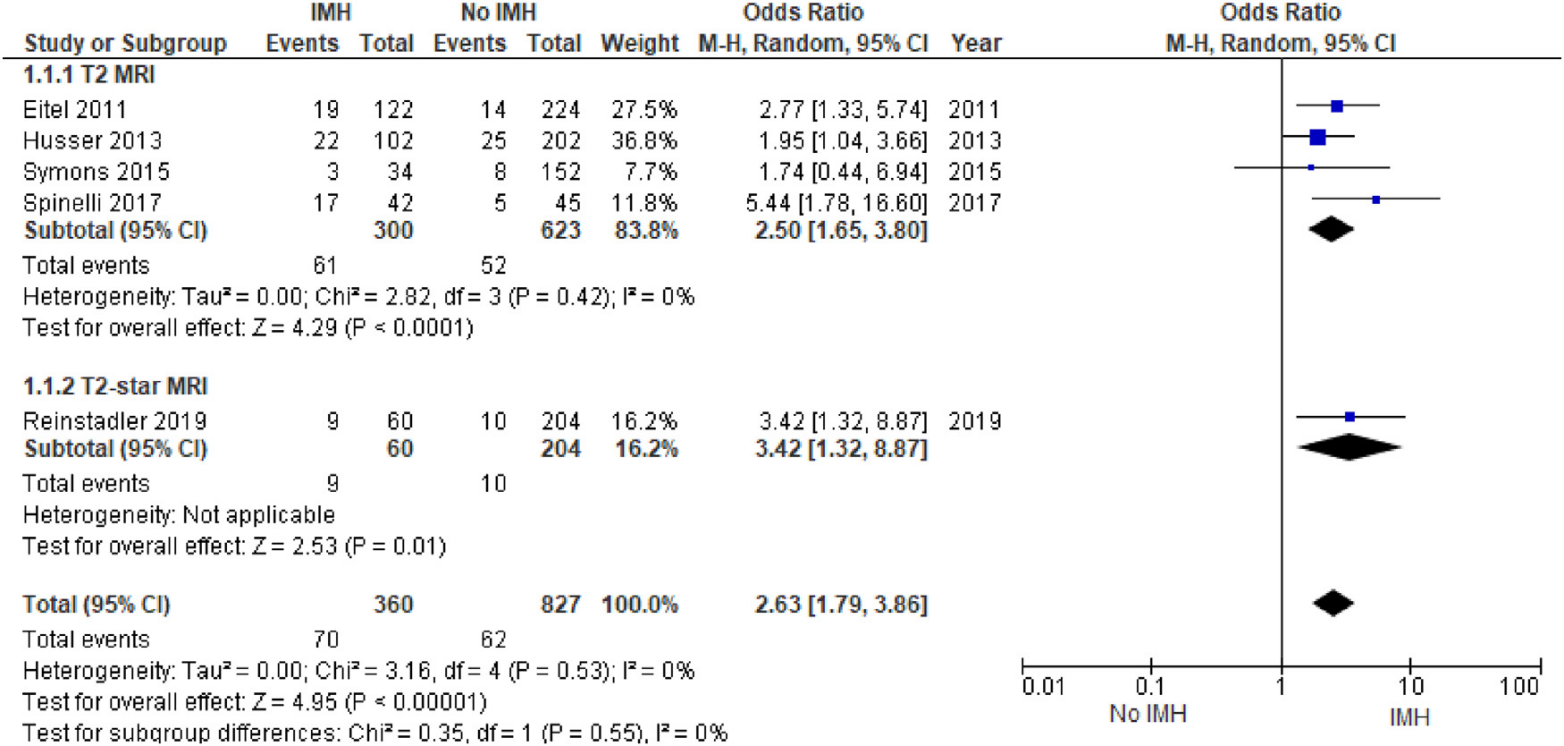
**Major adverse cardiovascular events.** IMH, intramyocardial hemorrhage; MRI, magnetic resonance imaging.

We were able to identify 5 studies comparing the rates of MACE. All the studies used similar definitions for MACE, defined as a composite of death, reinfarction, and new congestive heart failure after the index event. IMH was significantly associated with MACE (OR, 2.63; 95% CI, 1.79-3.86; *P* < .00001; I^2^ = 0%). There was no significant heterogeneity within this pooled analysis. The majority of these studies used multivariable analysis to determine that IMH is independently associated with MACE. In the subgroup analysis, there was a single study that used T2∗ MRI to assess for IMH; both subgroups showed a significant association of IMH with MACE (Figure 4).

**Figure 5:**
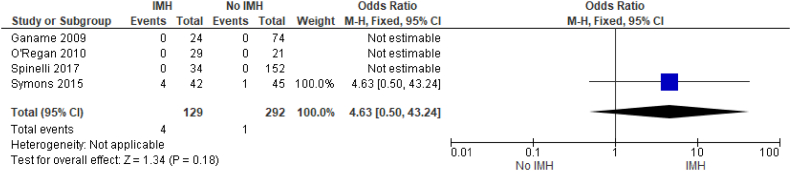
**Mortality.** IMH, intramyocardial hemorrhage.

There were limited available data regarding IMH and mortality with very few mortalities reported in these studies. From the available data, we found no significant difference in patients with IMH versus those without it (OR, 4.64; 95% CI, 0.50-43.24; *P* < .18) (Figure 5).

### Association of IMH with left ventricular remodeling variables and procedural variables

**Figure 6:**
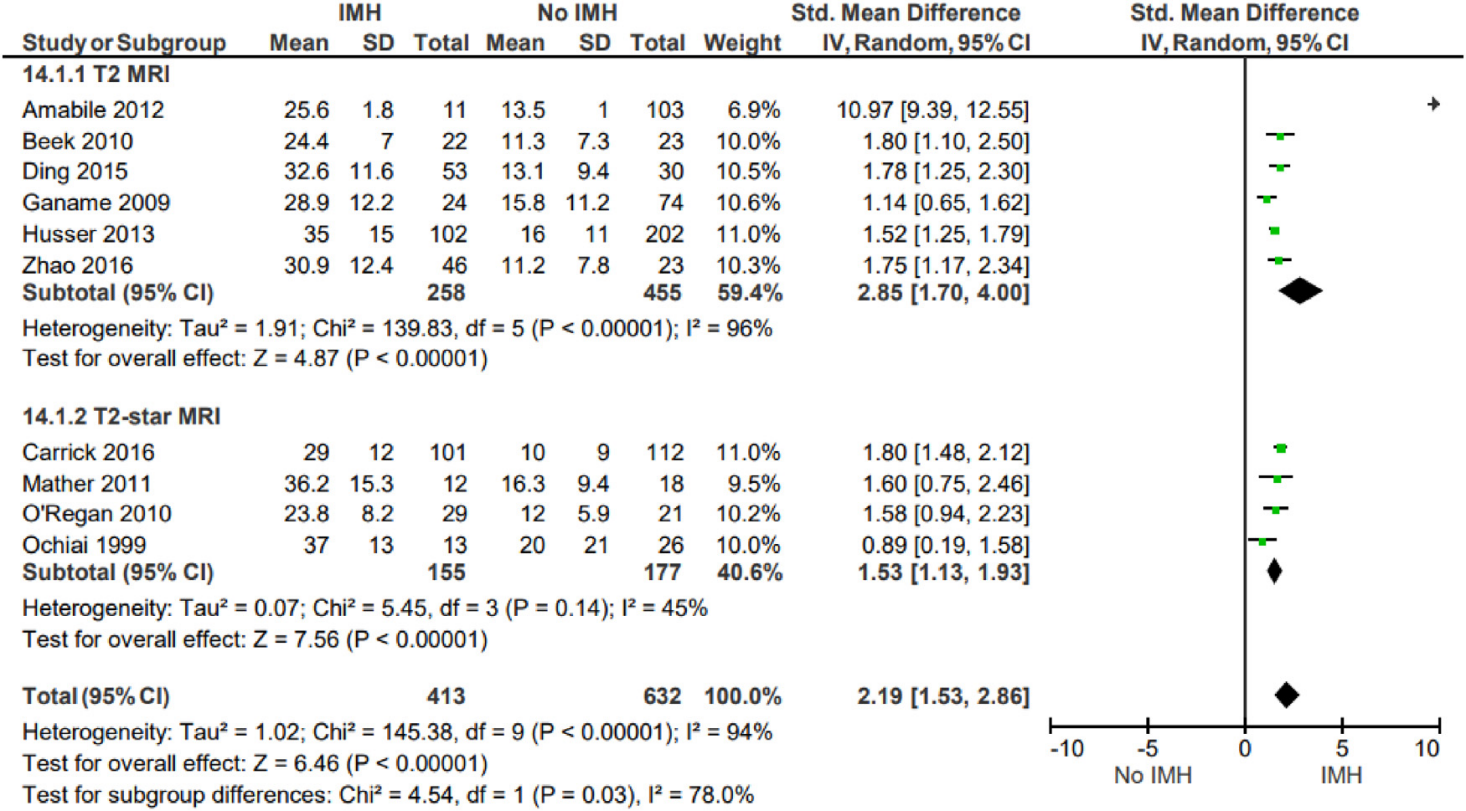
**Infarct size (% of the left ventricle).** IMH, intramyocardial hemorrhage; MRI, magnetic resonance imaging.

**Figure 7:**
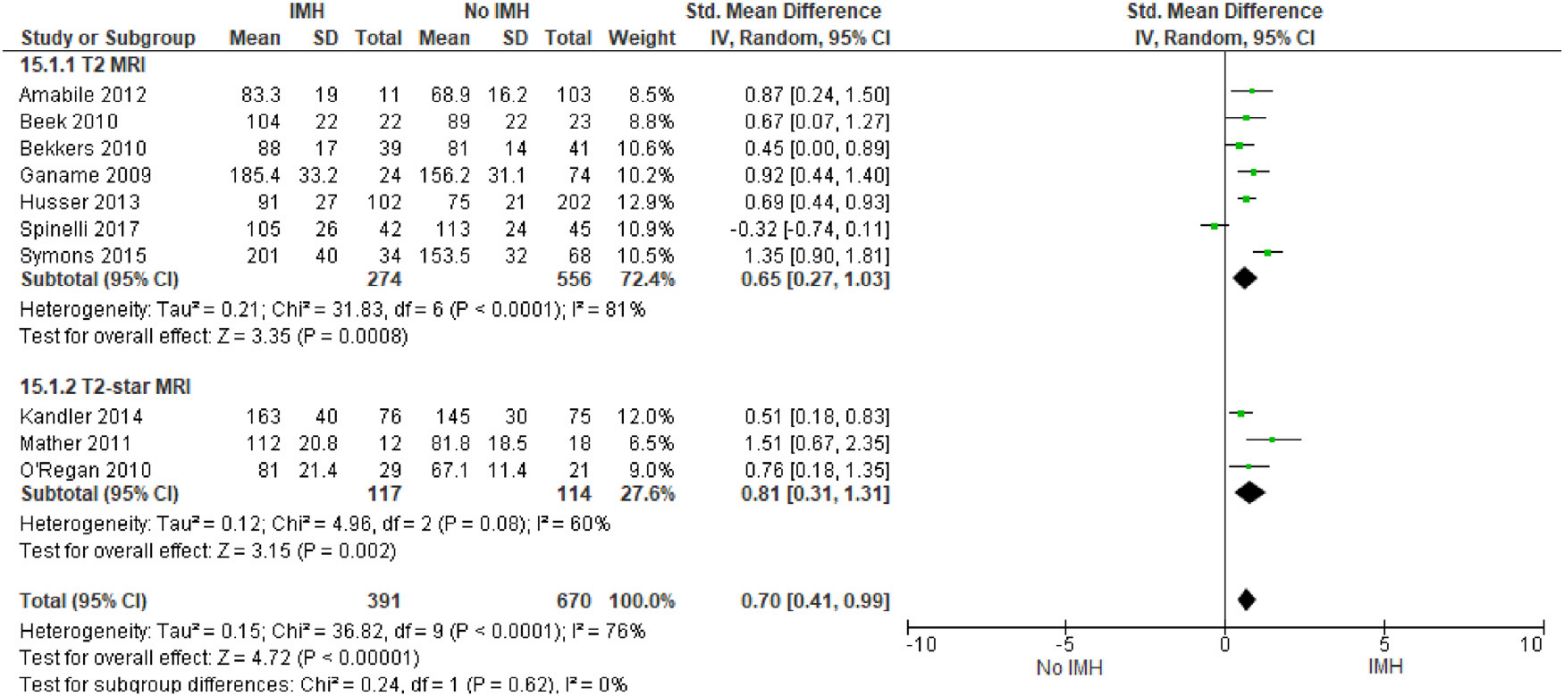
**Left ventricular end-diastolic volume (inmL).** IMH, intramyocardial hemorrhage; MRI, magnetic resonance imaging.

**Figure 8:**
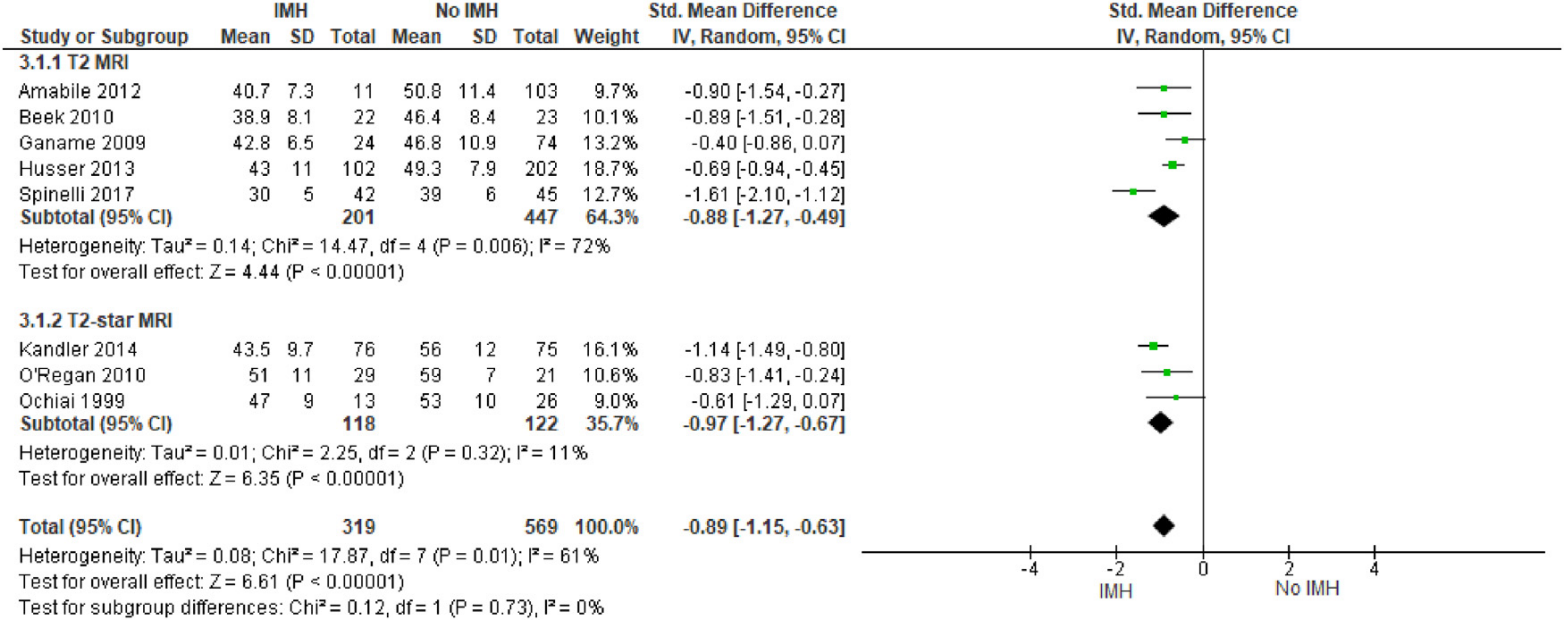
**Left ventricular ejection fraction.** IMH, intramyocardial hemorrhage; MRI, magnetic resonance imaging.

Intramyocardial hemorrhage correlates with adverse left ventricular remodeling. IMH is positively associated with infarct size (SMD, 2.19; 95% CI, 1.53-2.86; *P* < .00001; I^2^ = 94%) (Figure 6) and LVEDV (SMD, 0.7; 95% CI, 0.41-0.99; *P* < .00001; I^2^ = 76%) (Figure 7). There was also a negative association of IMH with LVEF (SMD, −0.89; 95% CI, −1.15 to −0.63; *P* = .010; I^2^ = 61%) (Figure 8). All these outcomes showed a significant degree of heterogeneity, possibly owing to the observational nature of the studies and variable study methodologies. Subgroup analyses for T2∗ MRI showed that IMH assessed via T2∗ MRI is also similarly associated with adverse left ventricular remodeling outcomes. The majority of these studies used multivariable analysis to determine that IMH is independently associated with adverse left ventricular remodeling variables.

**Figure 9:**
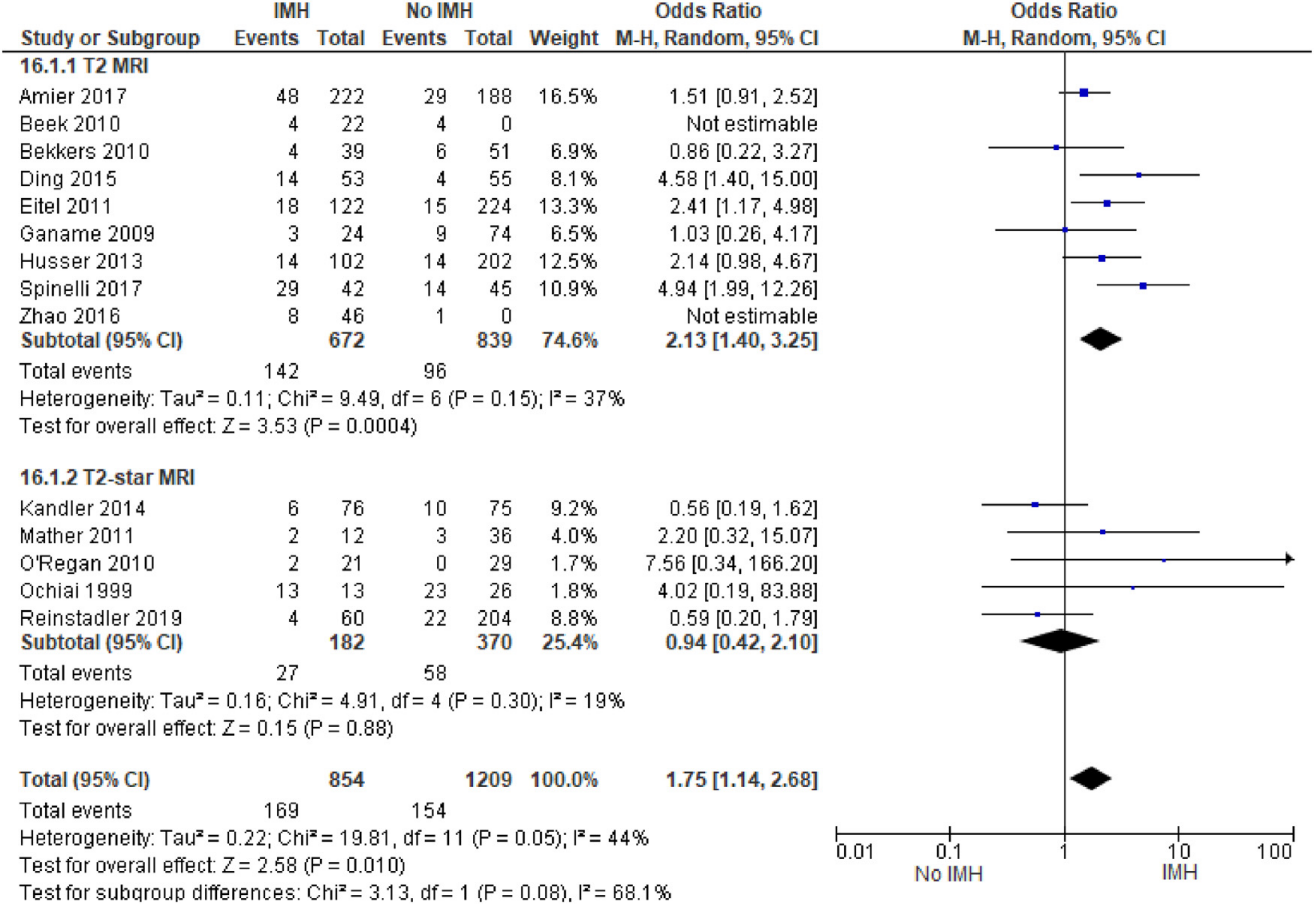
**Thrombolysis inMyocardialInfarction(TIMI)flow score of <3 after percutaneous coronary intervention.** IMH, intramyocardial hemorrhage; MRI, magnetic resonance imaging.

**Figure 10:**
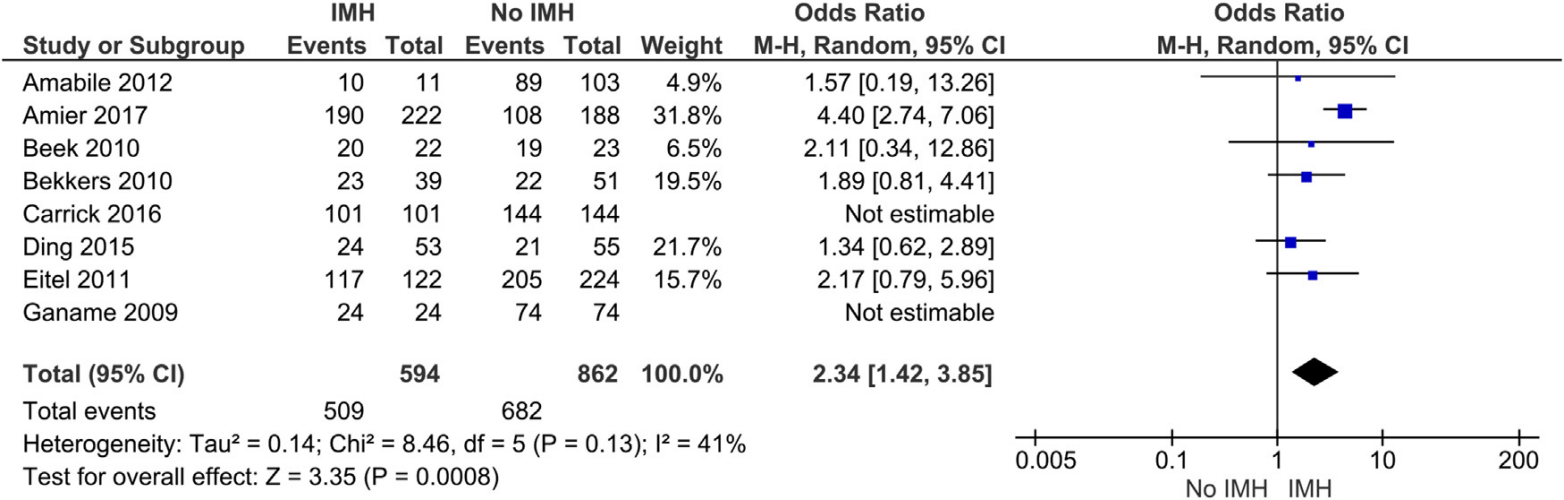
**GlycoproteinIIb/IIIa inhibitor.** IMH, intramyocardial hemorrhage.

We also found that IMH was significantly associated with having less than TIMI-3 flow after PCI (OR, 1.75; 95% CI, 1.14-2.68; *P* = .05; I^2^ = 44%) (Figure 9); notably, subgroup analysis of the studies that used T2∗ MRI showed no significant association between IMH and less than TIMI-3 flow after PCI. Interestingly, IMH is also significantly associated with the use of GP IIb/IIIa inhibitors (OR, 2.34; 95% CI, 1.42-3.85; *P* = .0008; I^2^ = 41%) (Figure 10). The T2∗ studies available for subgroup analysis under the GP IIb/IIIa inhibitor outcome were unable to be evaluated because of inestimable effect, thus subgroup analysis could not be performed.

**Figure 11:**
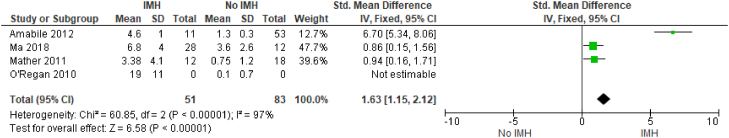
**Microvascular obstruction.** IMH, intramyocardial hemorrhage.

Finally, we evaluated the impact of IMH on the extent of MVO (reported as a percentage of the left ventricle). We found that IMH is associated with a greater extent of MVO (SMD, 1.63; 95% CI, 1.15-2.12; *P* < .00001; I^2^ = 97%) (Figure 11).

## Discussion

The main findings from our systematic review and meta-analysis are as follows: (1) IMH is present in approximately 40% of the patients who experienced STEMI (Figure 3); (2) there is a significant correlation between IMH and adverse clinical outcomes; (3) a significant correlation exists between IMH and adverse left ventricular remodeling in the form of increased infarct size, increased LVEDV, and lower LVEF; and (4) male sex, smoking, and LAD infarcts were predictors of IMH. We also found certain procedural variables associated with IMH, namely reduced TIMI flow after PCI and the use of GP IIb/IIIa inhibitors (Central Illustration).

**Central Illustration:**
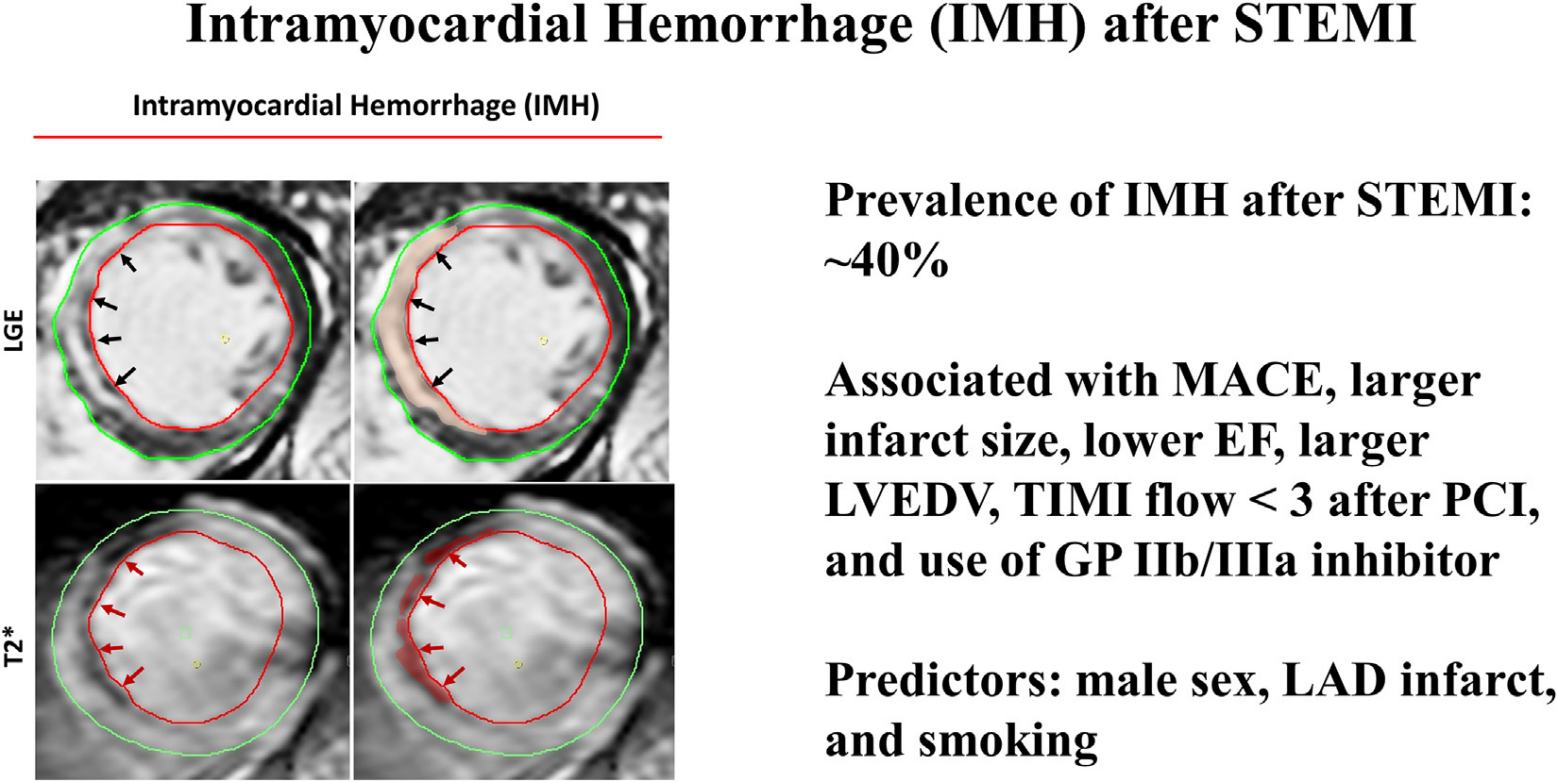
Intramyocardial hemorrhage after ST-elevation myocardial infarction. Prevalence of intramyocardial hemorrhage after ST-elevation myocardial infarction is approximately 40%. It is associated with major adverse cardiovascular events, larger infarct size, lower ejection fraction, larger left ventricular end-diastolic volume, thrombolysis in myocardial infarction flow score of <3 after percutaneous coronary intervention, and use of GP IIb/IIIa inhibitors. Predictors are male sex, anterior descending infarcts, and smoking.

We identified 5 studies investigating the association between IMH and MACE for a total of 1187 patients and found that IMH is a predictor of MACE (Figure 4). This is in agreement with findings from the previous smaller observational studies. The majority of these studies demonstrated a significant association between IMH and MACE but were limited by the small sample size. Most recently, in a univariate and multivariate analysis by Reinstadler et al,^22^ IMH was found to be an independent predictor of MACE, with multiple prior observational studies having noted similar findings.^17^^,^^18^^,^^25^ Of the included studies, Symons et al^25^ did not demonstrate a significant association between IMH and MACE (OR, 1.74; 95% CI, 0.44-6.94); notably, clinical follow-up for adverse events in that study was done only for 4 months, whereas the remaining studies followed up for MACE for at least 6 months. In all included studies, MACE was defined as repeat infarct, hospitalization for heart failure after the index event, or death.

No significant association was seen between IMH and mortality in our study, likely owing to the small number of deaths reported in these studies (Figure 5). Importantly, patients who experienced STEMI who were too unstable for CMR imaging, or those with early mortalities, were either excluded or unable to be evaluated in these studies. Therefore, the reported mortality rates in these cohort studies likely underestimate the true mortality rate in patients who experienced STEMI with IMH.

Our findings further demonstrate that IMH is associated with larger infarct size, larger LVEDV, and lower LVEF (Figure 6, Figure 7, Figure 8). Consistent with our results, multiple prior and smaller studies have noted that IMH is associated with adverse left ventricle remodeling after myocardial infarction. We identified 10 studies that evaluated the association between IMH and infarct size for a total of 1045 patients; all the included studies noted that IMH is associated with larger infarct sizes.

Similarly, we identified 10 studies that evaluated the association between IMH and LVEDV for a total of 1061 patients. Of the 10 studies, 9 showed a moderate impact of IMH with larger LVEDV. We identified 9 studies that evaluated the association between IMH and LVEF for a total of 888 patients; all 9 studies noted that IMH had a negative impact on LVEF.

An observational analysis by Kidambi et al^29^ has previously noted that recovery of contractile function was diminished in the presence of MVO and even more so in the presence of IMH; patients with IMH were also noted to have larger infarct zones. A prior echocardiogram substudy of the VALIANT trial noted that larger infarct size and decreased LVEF were predictive of all-cause mortality and increased LVEDV correlated with adverse clinical events.^30^ As such, the findings of our study further confirm the prognostic value of identification of IMH via CMR. Our review shows that patients with IMH tended to have a greater degree of MVO than those without IMH (Figure 11); IMH and MVO may be interrelated processes. Importantly, smaller studies have shown that IMH tends to be associated with a greater extent of MVO; our results are also consistent with this. Reinstadler et al^22^ is one of the few studies that compared the prognostic significance of IMH with other CMR variables such as MVO, infarct size, and ejection fraction. This analysis demonstrated that IMH is an independent predictor of adverse events, even when other CMR variables such as MVO are included in a multivariable model.^21^ Another recent study has reported clinical and experimental data demonstrating that IMH is not only a marker of large infarcts but actually worsens myocardial infarction and reduces myocardial salvage after reperfusion. This new work nicely complements this meta-analysis and indicates that IMH is a biological process that worsens myocardial infarction.^8^

Prior studies have noted poor TIMI flow before PCI as a risk factor for MVO. Similarly, we found that IMH is associated with worse TIMI flow after PCI (Figure 9). We identified 14 studies that examined the relationship between IMH and TIMI flow grade after PCI. Currently, there are no specific baseline parameters that are used to determine the risk of IMH. However, studies have shown associations between hyperglycemia, persistent ST-segment elevations, LAD artery infarctions, smoking,^31^ and low initial TIMI flow.^10^ Importantly, we found that LAD infarcts, male sex, and smoking history were significantly associated with IMH (Table 2).

There are several limitations to our meta-analysis. The included studies are observational in nature and are subject to selection and treatment bias. Selection bias may impact the results of the included studies because only patients deemed stable for CMR imaging would have undergone this imaging study. Subjects who died within the first few days of their myocardial infarction presentation or those with large infarcts, congestive heart failure, or cardiogenic shock were likely deemed too unstable for CMR. Therefore, these findings may be a conservative estimate of the prevalence and prognostic significance of IMH. Furthermore, inconsistent follow-up duration, variation of patient populations across the studies, and variation in disease severity on presentation may explain the degree of heterogeneity observed in our outcomes.

In addition, there was variability in the imaging techniques used to confirm the presence of IMH. Two studies used T2-weighted imaging to assess for IMH,^10^^,^^12^ 8 used T2-STIR,^11^^,^^13^^,^^14^^,^^16^, ^17^, ^18^^,^^25^^,^^26^ and 6 used T2∗-weighted imaging.^15^^,^^19^^,^^20^^,^^22^^,^^27^^,^^28^ Mather et al^21^ used T2- and T2∗-weighted imaging to confirm the presence of IMH. The study by Spinelli et al^24^ used speckle-tracking echocardiography, and T2-STIR CMR was used to confirm the presence of IMH in 7 of the 45 patients in this study.

T2 and T2∗ are the most frequently used CMR approaches to assess IMH. They have been shown to have greater diagnostic utility for detecting IMH than the T1 approach. In addition, recent studies have shown that T2∗ sequences are more reliable in detecting IMH.^32^, ^33^, ^34^ As such, we performed subgroup analyses for the majority of the selected outcomes. Regarding MACE, LVEF, infarct size, and LVEDV, subgroup analyses of studies that used T2∗ MRI showed findings similar to the overall analysis. Interestingly, among the studies that used T2∗ MRI, there was no statistically significant association between IMH and less than TIMI-3 flow after PCI. Regardless of the CMR imaging sequences used, our study demonstrates that IMH detected with CMR is an important prognostic factor for patients who experience STEMI.

Despite these limitations, our study has several strengths. To the best of our knowledge, this study presents the largest pooled analysis of patients with IMH in which T2-CMR imaging was used in the majority to evaluate patients who experience acute STEMI, with almost 3000 subjects. This provides a high level of evidence for the prevalence and clinical impact of IMH. Previously, Hamirani et al^7^ performed a meta-analysis examining the association between MVO between left ventricular remodeling and adverse clinical outcomes. Although the study provided strong results regarding MVO as a prognostic marker, their analysis regarding IMH was based on a limited data set of approximately 1000 subjects.^7^ Our study provides a much stronger result regarding IMH based on a larger pooled sample size, nearly triple the size of the Hamirani et al^7^ analysis. Adding to the strength of our study, we performed subgroup analyses for the majority of the assessed outcomes to determine whether there were any significant differences between T2∗ MRI and standard T2 imaging.

These findings have significant clinical implications, showing that CMR imaging has an important role in post-STEMI prognostication. Furthermore, a significant portion of STEMI patients have IMH. We found that approximately 40% of STEMI patients have evidence of IMH on CMR imaging. The cause of the detrimental effect of IMH remains an area of the ongoing investigation. A key factor may be prolonged inflammatory reaction due to toxic hemoglobin degradation products. This has been shown to affect scar remodeling and subsequent reduction in tensile strength and chamber dilatation.^28^ Persistent iron in the infarct zone has also been associated with death or heart failure.^29^ IMH may prove to be a modifiable process, although there is no specific therapy targeting IMH at this time.^15^ Arheden^35^ noted that measurement of IMH could be integral in assessing the results of different STEMI treatment regimens.^36^ Therapy in the future could be tailored to reduce the degree of IMH by adjusting antithrombotic regimens. Notably, we also found a significant association between IMH and the use of GP IIb/IIIa inhibitors (Figure 10); similar findings were noted in the study by Amier et al.^12^ Prior studies of GP IIb/IIIa inhibitors have demonstrated benefit in STEMI; however, they are associated with a higher risk of bleeding.^37^ Whether periprocedural antiplatelet and antithrombotic medications can be tailored to minimize IMH is an area for future investigation.

Although hemorrhagic transformation in acute stroke is a well-recognized phenomenon, there has been comparably little attention to IMH after STEMI. Imaging identification of hemorrhagic transformation within the infarct tissue is a key step toward accurate and effective clinical management. Novel therapeutic strategies may be required for hemorrhagic infarcts.^38^

## Conclusion

In conclusion, our study shows that approximately 40% of patients who experience STEMI have evidence of IMH. IMH is a predictor of adverse clinical outcomes and adverse left ventricular remodeling in patients who experience STEMI. CMR imaging has an important role in the identification of these patients. Detection and quantification of IMH by CMR may be useful as a surrogate end point in clinical trials investigating novel postmyocardial infarction therapeutics. Future studies may investigate novel therapeutic strategies for minimizing IMH or improving the resolution of IMH.

## Footnotes

To access the supplementary material accompanying this article, visit the online version of the *Journal of the Society for Cardiovascular Angiography & Interventions* at 10.1016/j.jscai.2022.100444.
