# Supplementary material for: Impact of Intramyocardial Hemorrhage on Clinical Outcomes in ST-Elevation Myocardial Infarction: A Systematic Review and Meta-analysis
Source: J Soc Cardiovasc Angiogr Interv. 2022 Aug 26;1(6):100444. doi: 10.1016/j.jscai.2022.100444 (PMC11307811; doi:10.1016/j.jscai.2022.100444)
Supplement: Supplementary File [file mmc1.docx]

Supplementary Material

Search Strategy

**PubMed/MEDLINE**

Query: intramyocardial hemorrhage STEMI NOT ("case reports"[Publication Type] OR "editorial"[Publication Type] OR "guideline"[Publication Type] OR "introductory journal article"[Publication Type] OR "meta analysis"[Publication Type] OR "news"[Publication Type] OR "practice guideline"[Publication Type] OR "review"[Publication Type] OR "systematic review"[Publication Type])

Dates: Inception – 04/13/2021

Total: 57 articles

**Embase**

Query: intramyocardial hemorrhage STEMI NOT ('animal cell'/de OR 'animal experiment'/de OR 'animal model'/de OR 'animal tissue'/de OR 'case report'/de OR 'clinical protocol'/de OR 'diagnostic test accuracy study'/de OR 'meta analysis'/de OR 'methodology'/de OR 'nonhuman'/de OR 'practice guideline'/de OR 'questionnaire'/de OR 'systematic review'/de OR 'chapter'/it OR 'editorial'/it OR 'erratum'/it OR 'note'/it OR 'review'/it OR 'short survey'/it)

Dates: Inception – 04/13/2021

Total: 48 articles

**COCHRANE LIBRARY**

Query: intramyocardial hemorrhage STEMI

Dates: Inception – 04/13/2021

Total: 8 articles

**Supplementary Table 1:** PRISMA Checklist

| **Section/topic** | **#** | **Checklist item** | **Reported on page #** |
| --- | --- | --- | --- |
| **TITLE** | | |  |
| Title | 1 | Identify the report as a systematic review, meta-analysis, or both. | 1 |
| **ABSTRACT** | | |  |
| Structured summary | 2 | Provide a structured summary including, as applicable: background; objectives; data sources; study eligibility criteria, participants, and interventions; study appraisal and synthesis methods; results; limitations; conclusions and implications of key findings; systematic review registration number. | 2 |
| **INTRODUCTION** | | |  |
| Rationale | 3 | Describe the rationale for the review in the context of what is already known. | 3 |
| Objectives | 4 | Provide an explicit statement of questions being addressed with reference to participants, interventions, comparisons, outcomes, and study design (PICOS). | 3 |
| **METHODS** | | |  |
| Protocol and registration | 5 | Indicate if a review protocol exists, if and where it can be accessed (e.g., Web address), and, if available, provide registration information including registration number. | NA |
| Eligibility criteria | 6 | Specify study characteristics (e.g., PICOS, length of follow-up) and report characteristics (e.g., years considered, language, publication status) used as criteria for eligibility, giving rationale. | 3,4 |
| Information sources | 7 | Describe all information sources (e.g., databases with dates of coverage, contact with study authors to identify additional studies) in the search and date last searched. | 3,4 |
| Search | 8 | Present full electronic search strategy for at least one database, including any limits used, such that it could be repeated. | Supplementary page 2 |
| Study selection | 9 | State the process for selecting studies (i.e., screening, eligibility, included in systematic review, and, if applicable, included in the meta-analysis). | Figure 1 |
| Data collection process | 10 | Describe method of data extraction from reports (e.g., piloted forms, independently, in duplicate) and any processes for obtaining and confirming data from investigators. | 3,4 |
| Data items | 11 | List and define all variables for which data were sought (e.g., PICOS, funding sources) and any assumptions and simplifications made. | 3,4 |
| Risk of bias in individual studies | 12 | Describe methods used for assessing risk of bias of individual studies (including specification of whether this was done at the study or outcome level), and how this information is to be used in any data synthesis. | 3,4 |
| Summary measures | 13 | State the principal summary measures (e.g., risk ratio, difference in means). | 3,4 |
| Synthesis of results | 14 | Describe the methods of handling data and combining results of studies, if done, including measures of consistency (e.g., I^2^) for each meta-analysis. | 3,4 |

| **Section/topic** | **#** | **Checklist item** | **Reported on page #** |
| --- | --- | --- | --- |
| Risk of bias across studies | 15 | Specify any assessment of risk of bias that may affect the cumulative evidence (e.g., publication bias, selective reporting within studies). | 3,4 |
| Additional analyses | 16 | Describe methods of additional analyses (e.g., sensitivity or subgroup analyses, meta-regression), if done, indicating which were pre-specified. | 3,4 |
| **RESULTS** | | |  |
| Study selection | 17 | Give numbers of studies screened, assessed for eligibility, and included in the review, with reasons for exclusions at each stage, ideally with a flow diagram. | Figure 1 |
| Study characteristics | 18 | For each study, present characteristics for which data were extracted (e.g., study size, PICOS, follow-up period) and provide the citations. | Table 2 and 3 |
| Risk of bias within studies | 19 | Present data on risk of bias of each study and, if available, any outcome level assessment (see item 12). | Supplement page 5 |
| Results of individual studies | 20 | For all outcomes considered (benefits or harms), present, for each study: (a) simple summary data for each intervention group (b) effect estimates and confidence intervals, ideally with a forest plot. | 5-8 |
| Synthesis of results | 21 | Present results of each meta-analysis done, including confidence intervals and measures of consistency. | 5-8 |
| Risk of bias across studies | 22 | Present results of any assessment of risk of bias across studies (see Item 15). | 5-8 |
| Additional analysis | 23 | Give results of additional analyses, if done (e.g., sensitivity or subgroup analyses, meta-regression [see Item 16]). | 5-8 |
| **DISCUSSION** | | |  |
| Summary of evidence | 24 | Summarize the main findings including the strength of evidence for each main outcome; consider their relevance to key groups (e.g., healthcare providers, users, and policy makers). | 5-8 |
| Limitations | 25 | Discuss limitations at study and outcome level (e.g., risk of bias), and at review-level (e.g., incomplete retrieval of identified research, reporting bias). | 5-8 |
| Conclusions | 26 | Provide a general interpretation of the results in the context of other evidence, and implications for future research. | 5-8 |
| **FUNDING** | | |  |
| Funding | 27 | Describe sources of funding for the systematic review and other support (e.g., supply of data); role of funders for the systematic review. | 9 |

**Supplementary Table 2:** The Newcastle-Ottawa Scale (NOS) for assessing the quality of nonrandomized studies in meta-analyses

| Newcastle-Ottawa Scale (NOS) for assessing the quality of nonrandomised studies in meta-analyses | | | | | | | | | |
| --- | --- | --- | --- | --- | --- | --- | --- | --- | --- |
| Study | Selection of cohorts (max 1 star) | | | | Comparability of cohorts (max 2 stars) | Outcome (max 1 star) | | | Total score |
|  | Representativeness of the IMH cohort | Selection of the no IMH cohort | Ascertainment of exposure | Demonstration that outcome of interest was not present at start of study | Comparability of cohorts on the basis of the design or analysis | Assessment of outcome | Was follow up long enough for outcomes to occur | Adequacy of follow up of cohorts |  |
| Amabile 2012 | * | * | * |  | ** | * | * | * | 8 |
| Amier 2017 | * | * | * |  | * | * | * | * | 7 |
| Beek 2010 | * | * | * | * | ** | * | * |  | 8 |
| Bekkers 2010 | * | * | * | * | ** | * | * | * | 9 |
| Carrick 2016 | * | * | * |  | * | * | * | * | 7 |
| Ding 2015 | * | * | * |  | ** | * | * | * | 8 |
| Eitel 2011 | * | * | * |  | ** | * | * | * | 8 |
| Ganame 2009 | * | * | * |  | * | * | * | * | 7 |
| Husser 2013 | * | * | * | * | * | * | * | * | 8 |
| Kandler 2014 | * | * | * |  | * | * | * | * | 7 |
| Ma 2018 | * | * | * | * | * | * | * | * | 8 |
| Mather 2011 | * | * | * | * | * | * | * | * | 8 |
| O'Regan 2010 | * | * | * | * | ** | * | * | * | 9 |
| Ochiai 1999 | * | * | * | * | ** | * | * |  | 8 |
| Reinstadler 2019 | * | * | * |  | ** | * | * | * | 8 |
| Spinelli 2017 | * | * | * |  | ** | * | * | * | 8 |
| Symons 2015 | * | * | * | * | * | * | * | * | 8 |
| Zhao 2016 | * | * | * | * | ** | * | * | * | 9 |

Each asterisk represents one star in the Newcastle-Ottawa Scaling System (NOS). The maximum stars are 2 for comparability and 1 are for all other categories. Each star counts towards the total score. Score of 5 to 6 considered as moderate quality and 7 to 9 as high quality.

**Supplementary Figure 1:** Funnel plot for assessment of publication bias comparing IMH with no IMH regarding MACE.

**
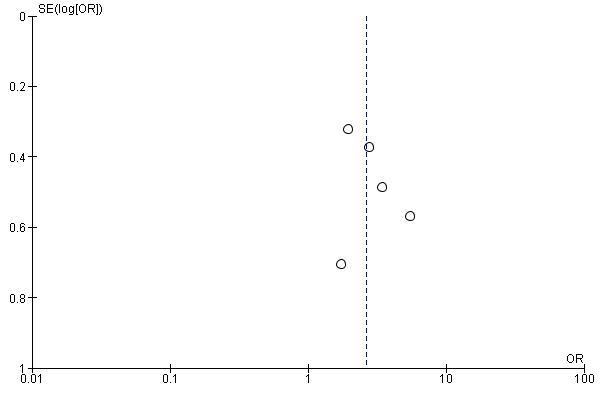
**

Note that there is no evident asymmetry of the points.

SE: standard error, OR: Odds ratio

**Supplementary Figure 2:** Funnel plot for assessment of publication bias comparing IMH with no IMH regarding infarct size.

**
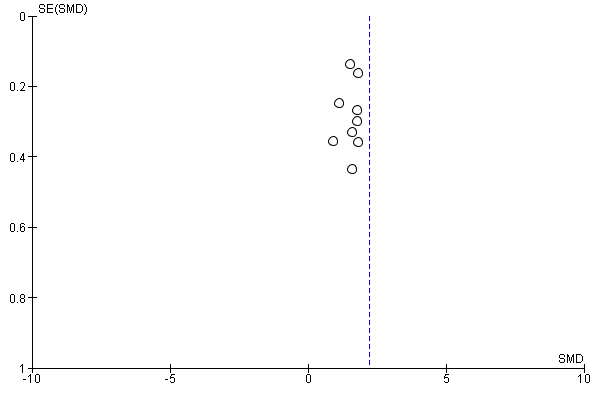
**

Note that there is no evident asymmetry of the points.

SE: standard error, OR: Odds ratio

**Supplementary Figure 3:** Funnel plot for assessment of publication bias comparing IMH with no IMH regarding TIMI flow less than 3 post-PCI.

**
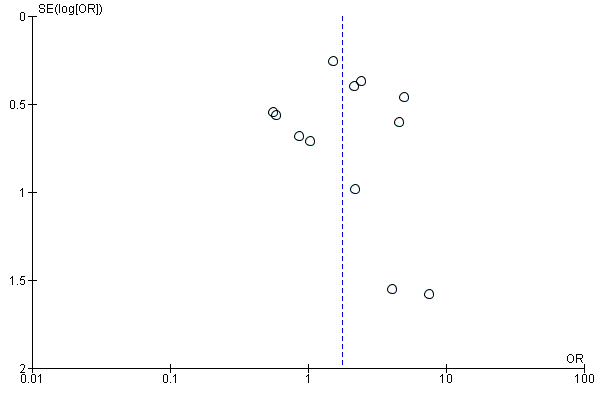
**

Note that there is no evident asymmetry of the points.

SE: standard error, OR: Odds ratio

**Supplementary Figure 4:** Funnel plot for assessment of publication bias comparing IMH with no IMH regarding LVEF.

**
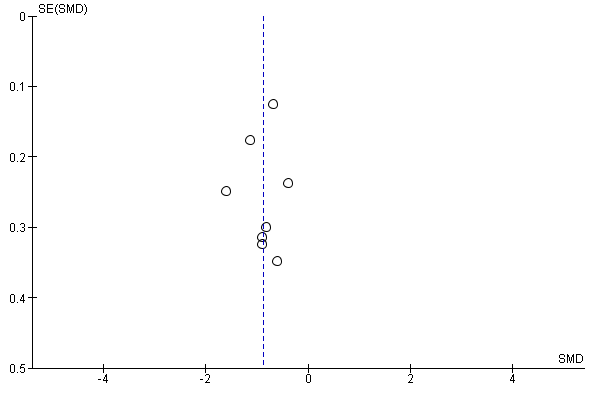
**

Note that there is no evident asymmetry of the points.

SE: standard error, OR: Odds ratio
